# Value of Perfusion-Weighted MR Imaging in the Assessment of Early Cerebral Alterations in Neurologically Asymptomatic HIV-1-Positive and HCV-Positive Patients

**DOI:** 10.1371/journal.pone.0102214

**Published:** 2014-07-11

**Authors:** Joanna Bladowska, Brygida Knysz, Anna Zimny, Krzysztof Małyszczak, Anna Kołtowska, Paweł Szewczyk, Jacek Gąsiorowski, Michał Furdal, Marek J. Sąsiadek

**Affiliations:** 1 Department of General Radiology, Interventional Radiology and Neuroradiology, Chair of Radiology, Wroclaw Medical University, Wroclaw, Poland; 2 Department of Infectious Diseases, Liver Diseases and Acquired Immune Deficiency, Wroclaw Medical University, Wroclaw, Poland; 3 Division of Psychotherapy and Psychosomatic Medicine, Department of Psychiatry, Wroclaw Medical University, Wrocław, Poland; 4 Regional Specialistic Hospital, Department of Cardiology, Wroclaw, Poland; University of California, San Francisco, United States of America

## Abstract

**Background and Purpose:**

Asymptomatic central nervous system (CNS) involvement occurs in the early stage of the human immunodeficiency virus (HIV) infection. It has been documented that the hepatitis C virus (HCV) can replicate in the CNS. The aim of the study was to evaluate early disturbances in cerebral microcirculation using magnetic resonance (MR) perfusion-weighted imaging (PWI) in asymptomatic HIV-1-positive and HCV-positive patients, as well as to assess the correlation between PWI measurements and the clinical data.

**Materials and Methods:**

Fifty-six patients: 17 HIV-1-positive non-treated, 18 HIV-1-positive treated with combination antiretroviral therapy (cART), 7 HIV-1/HCV-positive non-treated, 14 HCV-positive before antiviral therapy and 18 control subjects were enrolled in the study. PWI was performed with a 1.5T MR unit using dynamic susceptibility contrast (DSC) method. Cerebral blood volume (CBV) measurements relative to cerebellum (rCBV) were evaluated in the posterior cingulated region (PCG), basal ganglia (BG), temporoparietal (TPC) and frontal cortices (FC), as well as in white matter of frontoparietal areas. Correlations of rCBV values with immunologic data and liver histology activity index (HAI) were analyzed.

**Results:**

Significantly lower rCBV values were found in the right TPC and left FC as well as in PCG in HIV-1-positive naïve (p = 0.009; p = 0.020; p = 0.012), HIV-1 cART treated (p = 0.007; p = 0.009; p = 0.033), HIV-1/HCV-positive (p = 0.007; p = 0.027; p = 0.045) and HCV-positive patients (p = 0.010; p = 0.005; p = 0.045) compared to controls. HIV-1-positive cART treated and HIV-1/HCV-positive patients demonstrated lower rCBV values in the right FC (p = 0.009; p = 0.032, respectively) and the left TPC (p = 0.036; p = 0.005, respectively), while HCV-positive subjects revealed lower rCBV values in the left TPC region (p = 0.003). We found significantly elevated rCBV values in BG in HCV-positive patients (p = 0.0002; p<0.0001) compared to controls as well as to all HIV-1-positive subjects. There were no significant correlations of rCBV values and CD4 T cell count or HAI score.

**Conclusions:**

PWI examination enables the assessment of HIV-related as well as HCV-related early cerebral dysfunction in asymptomatic subjects. HCV-infected patients seem to reveal the most pronounced perfusion changes.

## Introduction

It is well known that the human immunodeficiency virus (HIV) enters the brain soon after the onset of infection (within 3 to 6 days) [1], [2]. HIV crosses the blood-brain barrier through infected macrophages and microglia in a process called a ‘Trojan horse’ mechanism [3]. Due to this process the involvement of the central nervous system (CNS) in the course of HIV infection is already often observed in the early stage of the disease [1]–[5].

The hepatitis C virus (HCV) is a frequent co-infection with HIV as a result of similar routes of viral transmission [6], [7]. HIV/HCV co-infection is associated with worse clinical outcomes of both diseases in terms of systemic disease progression and mortality [8], [9].

It has been evidenced that HCV may invade the CNS as well, since that virus is not exclusively hepatotropic, as it can also replicate in leukocytes, including monocytes/macrophages. The infected monocytes may cross the blood-brain barrier and thus provide access of HCV into the CNS in a process similar to that described for HIV [1], [10]. Moreover, several studies have evidenced that HCV infection is associated with cognitive dysfunction, fatigue and depression [11]–[13].

Since both viruses mentioned above can infect the brain and impair CNS function, we hypothesize that they may cause alterations in cerebral perfusion, as measured by in vivo magnetic resonance (MR) perfusion weighted imaging (PWI). PWI is a method that brings information on cerebral flow at the capillary level (microvasculature) [14], [15]. Among a few PWI techniques, dynamic susceptibility contrast MR imaging (DSC-MRI) is the most often used one. DSC MRI enables noninvasive measurements of relative cerebral blood volume (rCBV), thus providing information similar to that obtained in PET and SPECT studies but with several advantages, such as lack of ionizing radiation, high spatial resolution and low relative cost [14], [15].

The aim of our study was to evaluate the early perfusion changes using PWI in the neurologically asymptomatic HIV-1-positive non-treated (naive), HIV-1-positive treated with combination antiretroviral therapy (cART), HIV-1/HCV-positive untreated with any antiviral drugs and HCV-positive patients before antiviral therapy, who presented a normal appearing brain on plain MR. The other purpose was also to evaluate correlation of the perfusion measurements with immunologic alterations in HIV-1-positive subjects as well as with the liver histology activity index (HAI) in HCV-infected patients.

It should be stressed that in the available literature there are only a few studies on the use of PWI in the assessment of early cerebral perfusion alterations in asymptomatic HIV-1-infected and HCV-infected patients with only mild liver disease [14], [16]–[18]. Furthermore, those previous studies did not evaluate the differences between HIV-1-positive non-treated, HIV-1-positive cART treated, HIV-1/HCV-positive and HCV-positive patients in one research [16], [17]. To the best of our knowledge this is the first report focusing on the analysis of early perfusion changes within normal appearing grey and white matter in a large group of various neurologically asymptomatic subjects.

## Materials and Methods

### Patients

Fifty-six neurologically asymptomatic patients: 17 HIV-1-positive who had no need of cART according to the recommendations of EACS [European Aids Clinical Society (*EACS*) Guidelines 2013, Version 7.0, 6–7] (4 women and 13 men; mean age 33 yrs), 18 HIV-1-positive receiving cART (8 women and 10 men; mean age 39.3 yrs), 7 HIV-1/HCV-positive (2 women and 5 men; mean age 36.6 yrs) before antiviral therapy for HIV and HCV infections, 14 HCV-positive naive patients (6 women and 8 men; mean age 38.2 yrs) and 18 normal control subjects (6 women and 12 men; mean age 34.69 yrs) were enrolled in the study. HIV-1-positive patients with HCV coinfection as well as HCV-positive subjects suffered from chronic viral hepatitis with HCV RNA positive results for longer than 6 months. In HIV-1-positive as well as HCV-positive patients plasma viral load (HIV RNA or HCV RNA) at the time of imaging was determined. The Cobas TaqMan test version 2.0 with High Pure system isolation, the Roche Diagnostics real-time PCR method for HIV or HCV was used. In all the HCV patients, a liver biopsy was performed. Histopathological examinations of the samples revealed HAI (histology activity index) inflammation score and HAI fibrosis score of 0–2, which means only mild liver disease.

The clinical characteristics of the studied groups are shown in Table 1.

**Table 1:** Clinical characteristics of studied patients.

Subjects	HIV-1 naive	HIV cART treated	HIV/HCV naive	HCV before treatment Genotype 1
Mean age (years)	33.0	39.3	36.6	38.2
IDU*	6 (28,6%)	6 (30%)	7 (77,8%)	0 (0%)
CD4a T cell count at imaging (cells/µl)	369–986	201–1040	413–1014	-
mean	499	599	580	
CD4n T cell (nadir) count (cells/µl)	300–986	3–462	10–779	-
mean	486	121	441	
HIV RNA at imaging (copies/ml)	3180–125000	<40	4440–68321	-
mean	23454	<40	4727	
HCV RNA at imaging (IU/ml)	-	-	-	58672–4485492
mean				1324553
cART** (years)	-	3.5–10.0	-	-
ADC stage*** (max.4)	0	0	0	0
Karnofsky score**** (max.100)	>80	>80	>80	>80

*IDU-intravenous drug users.

**cART-combination antiretroviral therapy.

***ADC stage-AIDS dementia complex stage.

****The Karnofsky Scale allows patients to be classified according to their functional impairment. The lower the Karnofsky score, the worse the survival for most serious illnesses.

### Ethics statement

The study was conducted in accordance with the guidelines of the local University Ethics Committee for conducting research involving humans. All participants provided their written informed consent to participate in this study, according to the Helsinki Declaration, and the study was approved by the Commission of Bioethics at Wroclaw Medical University (number of permission: KB-225/2008).

### Neurological evaluation

All patients underwent physical and neurological examinations, including determining their Karnofsky score (max. 100). The inclusion criteria were lack of neurological abnormalities in neurological examination and a Karnofsky score above 80. The exclusion criteria were as follows:

1. History of neurological diseases (e.g. inflammatory changes, brain neoplasms);
2. Psychiatric disease history (e.g. depression, schizophrenia, dementia).
3. Patients with CNS AIDS related events were excluded from the study.

### Psychological evaluation

Two cognitive tests: Wisconsin Card Sorting Test (WCST) as a measure of executive function and Brickenkamp’s d2 concentration endurance test as a measure of visual attention were used in order to assess possible deterioration of cognitive functions [19], [20].

### The control group (CG)

The healthy control group with negative anti-HIV and anti-HCV antibodies had no history of drug abuse or liver disease and consisted mainly of the hospital staff from the Department of Radiology and Department of Infectious Diseases.

### MR imaging protocols

Imaging was performed with a 1.5T Signa Hdx scanner (GE Healthcare) using a 16-channel coil dedicated for head and spine imaging. Conventional sequences included: axial, sagittal and coronal T2-weighted images, axial T1-weighted and FLAIR (fluid-attenuated inversion recovery sequence) images, as well as diffusion-weighted imaging (DWI). Only those subjects who had normal signal intensity of gray and white matter in all sequences mentioned above, without evidence of cerebral atrophy were included in our study.

### Perfusion weighted imaging (PWI)

PWI was performed with a DSC method, using a gradient-recalled T2*-weighted echo-planar imaging (EPI) sequence. The parameters used were as follows: TR/TE = 1900/80 ms, flip angle = 80°, number of excitations = 1, matrix size = 192×128, and slice thickness = 8 mm (with no gap). Perfusion images were obtained in axial slices parallel to anterior commissure – posterior commissure (AC-PC) line. Image acquisition started 10 seconds before contrast agent injection to establish a pre-contrast baseline. Ten seconds after the start of image acquisition, 0.2 mmol/kg of body weight gadopentetate dimeglumine (MultiHance, Bracco, Germany) was injected with a power injector (Medrad) at a rate of 5 mL/s through an intravenous catheter placed in the antecubital vein. This was immediately followed by a bolus injection of saline (20 mL at 5 mL/s). Total duration of acquisition was 1 minute and 24 seconds.

**Figure 1:**
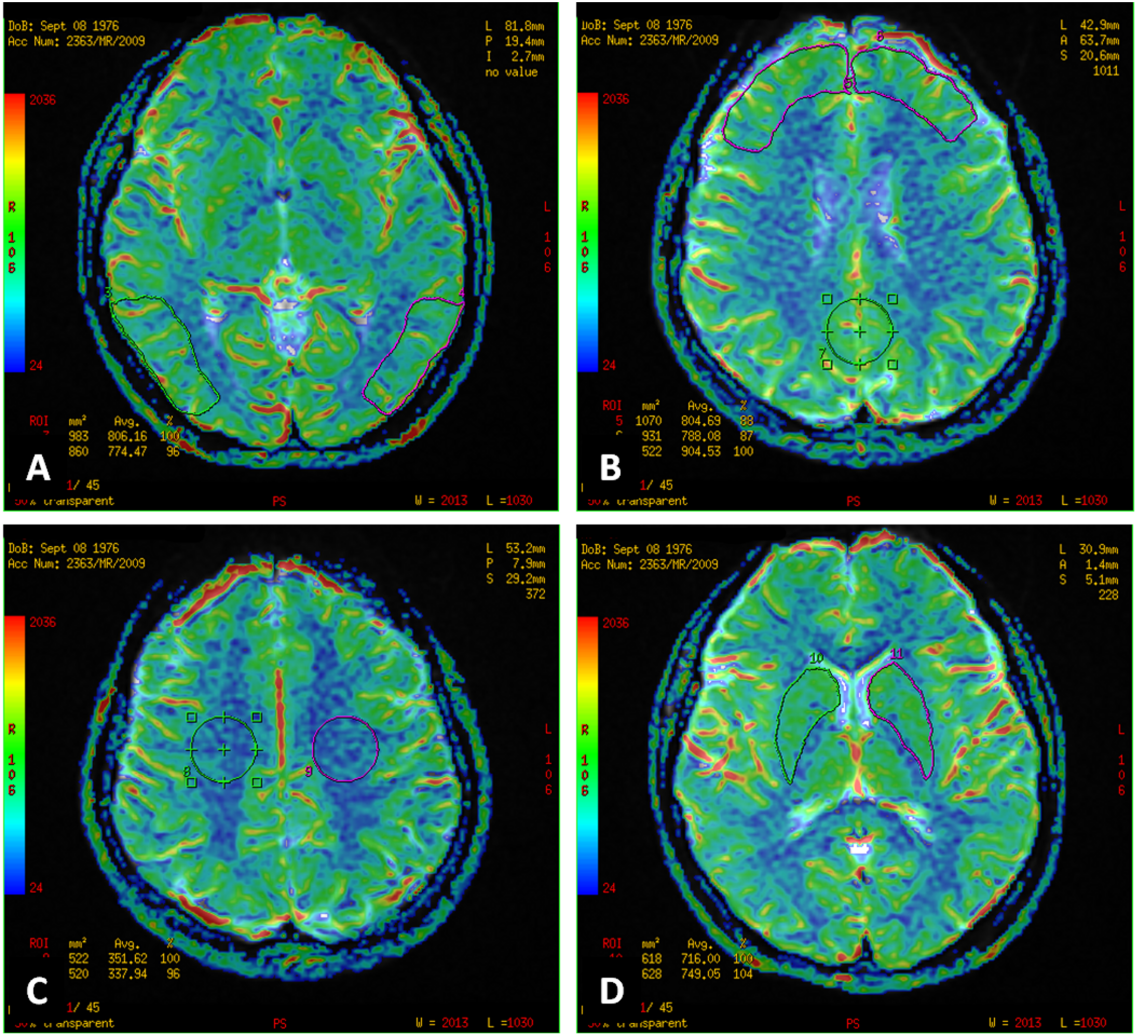
Perfusion-weighted MR examination. Regions of interest (ROI) on a perfusion map of cerebral blood volume (CBV) were located in the following regions: bilateral temporoparietal cortices (**A**), bilateral frontal association cortices and the posterior cingulate gyrus region (**B**), bilateral frontoparietal white matter (**C**), bilateral basal ganglia regions (**D**).

The dynamic images were post-processed using Functool software (GE Healthcare, ADW 4.4). CBV maps were computed on a pixel-wise basis from the first–pass data as described by Belliveau et al. [21]. All rCBV values were normalised to the mean values in the cerebellar cortex, which is the region minimally affected in patients with neurocognitive disorders as reported in the literature [22]. CBV values were obtained from several regions of interest (ROIs) and were placed manually in both cerebellar hemispheres (circular ROIs, size 525 mm^2^), temporoparietal cortices (8 mm above AP-CP), frontal cortices (16 mm above AP-CP), the posterior cingulate gyri (PCG) region (single circular ROI, size 525 mm^2^, covering cortex of the right and left cingulate gyrus), frontoparietal white matter in the centrum semiovale (circular ROIs, size 525 mm^2^) and the basal ganglia regions (irregular hand-drawn ROIs outlining the putamen, globus pallidus and caudate nucleus, size 500–600 mm^2^) (Fig. 1). Temporoparietal and frontal ROIs were almost rectangular in shape (size 800–1000 mm^2^).

### Statistical analysis

Analysis of variance (ANOVA) followed by the post hoc Tukey least significant difference (LSD) test was used for statistical evaluation of the results. Correlations between rCBV measurements and the immunologic data (CD4a, CD4n T cell count) in HIV-1-positive patients as well as the liver histology activity index (HAI) in HCV-positive subjects were estimated using Pearson’s correlation coefficients. Statistical computations were performed using the Statistica PL software package version 10.0, and p<0.05 was set as a significant level.

Additionally, corrections for multiple comparisons were performed. Applying the Bonferroni correction the significant p-values were established as p<0.0055 (p<0.05/n, where n = 9 variables/testing hypotheses: 9 rCBV measurements).

## Results

### Cognitive tests

There were no statistically significant differences in executive functions measured with all the WCST scales, but HCV patients performed the d2 attention task significantly slower than controls (variable TN, t-test, p = 0.03) and had lower values of speed-accuracy variable (TN-E, t-test, p = 0.02).

### Comparison of rCBV values between HIV-1 and HCV patients and control group (CG)

Compared to controls, HIV-1 naive patients showed significantly reduced rCBV values in the right temporoparietal cortex (p = 0.009), left frontal cortex (p = 0.020), as well as in posterior cingulate gyrus (p = 0.012) (table 2).

**Table 2:** Mean values and standard deviations (SD) of rCBV values in different brain regions in HIV-1 as well as HCV-infected patients compared to the control group (CG) with the results of the post-hoc Tukey LSD test.

Regions	HIV-1 naive	HIV-1 cART treated	HIV-1/HCV naive	HCV naive	CG
Right temporoparietal cortex (TPC)	1.17 (0.13)	1.17 (0.12)	1.13 (0.09)	1.16 (0.09)	1.27 (0.11)
*p values*	***0.009*** a	***0.007*** a	***0.007*** a	***0.010*** a	
Left temporoparietal cortex (TPC)	1.20 (0.10)	1.18 (0.12)	1.13 (0.07)	1.15 (0.08)	1.26 (0.11)
*p values*	*0.106*	***0.036*** a	***0.005*** a **^,^** b	***0.003*** a **^,^** b	
Right frontal cortex (FC)	1.18 (0.20)	1.12 (0.12)	1.11 (0.07)	1.14 (0.12)	1.26 (0.12)
*p values*	*0.209*	***0.009*** a	***0.032*** a	*0.053*	
Left frontal cortex (FC)	1.12 (0.21)	1.11 (0.12)	1.09 (0.05)	1.09 (0.11)	1.26 (0.13)
*p values*	***0.020*** a	***0.009*** a	***0.027*** a	***0.005*** a **^,^** b	
Posterior cingulate gyri (PCG)	1.25 (0.18)	1.27 (0.11)	1.24 (0.12)	1.27 (0.10)	1.38 (0.16)
*p values*	***0.012*** a	***0.033*** a	***0.045*** a	***0.045*** a	
Right frontoparietal white matter (FPWM)	0.52 (0.07)	0.54 (0.07)	0.55 (0.03)	0.52 (0.05)	0.56 (0.06)
*p values*	*0.061*	*0.337*	*0.686*	*0.080*	
Left frontoparietal white matter (FPWM)	0.54 (0.07)	0.54 (0.07)	0.55 (0.03)	0.52 (0.06)	0.56 (0.07)
*p values*	*0.380*	*0.449*	*0.871*	*0.193*	
Right basal ganglia (BG)	1.07 (0.13)	1.07 (0.10)	1.08 (0.11)	1.27 (0.09)	1.11 (0.11)
*p values*	*0.270*	*0.188*	*0.419*	***0.0002*** a **^,^** b	
Left basal ganglia (BG)	1.02 (0.11)	1.07 (0.11)	1.05 (0.09)	1.29 (0.12)	1.09 (0.10)
*p values*	*0.081*	*0.594*	*0.475*	***<0.0001*** a **^,^** b	

a statistically significant changes (p<0.05).

b statistically significant changes with the Bonferroni correction (p<0.0055).

HIV-1cART treated as well as HIV-1/HCV naive patients demonstrated a significant decrease of rCBV values in both temporoparietal cortices (right: p = 0.007; left: p = 0.036 and right: p = 0.007; left: p = 0.005, respectively), both frontal cortices (right: p = 0.009; left: p = 0.0009 and right: p = 0.032; left: p = 0.027, respectively), as well as in posterior cingulate gyrus (p = 0.033 and p = 0.045, respectively).

In HCV-infected patients rCBV values were significantly reduced compared to controls in both temporoparietal cortices (right: p = 0.010; left: p = 0.003), in the left frontal cortex (p = 0.005), as well as in posterior cingulate gyrus (p = 0.045).

In contrast, in both BG regions HCV subjects showed significantly elevated rCBV values compared to CG (right: p = 0.0002; left: p = <0.0001), as well as to HIV-1 naive, HIV-1cART treated and HIV-1/HCV naive patients (table 3).

**Table 3:** Comparisons of rCBV measurements in the basal ganglia regions among HIV-1 naive, HIV-1 cART treated, HIV-1/HCV and HCV naive patients with the results of the post hoc Tukey LSD test (p values).

Location	G1 vs G2	G1 vs G3	G1 vs G4	G2 vs G3	G2 vs G4	G3 vs G4
**Right basal ganglia (BG)**	0.844	0.976	**<0.0001** a **^,^** b	0.857	**<0.0001** a **^,^** b	**0.0003** a **^,^** b
**Left basal ganglia (BG)**	0.218	0.537	**<0.0001** a **^,^** b	0.752	**<0.0001** a **^,^** b	**<0.0001** a **^,^** b

G1-the group of HIV-1 positive non-treated (naive) patients.

G2-the group of HIV-1 positive cART treated patients.

G3-the group of HIV-1/HCV-positive non-treated (naive) patients.

G4-the group of HCV-positive non-treated (naive) patients.

a statistically significant changes (p<0.05).

b statistically significant changes with the Bonferroni correction (p<0.0055).

There were no significant differences in rCBV values among the studied groups in both frontoparietal white matter regions (table 2).

### Correlations of PWI measurements with the immunologic data in HIV-1 subjects

Correlations of rCBV values with the CD4a and CD4n T cell count were assessed for all HIV-1-positive and HIV-1/HCV-positive subjects enrolled in the study.

We found no statistically significant correlations between rCBV values in all evaluated regions and CD4a as well as CD4n T cell count.

### Correlations of PWI measurements with the histological data in HCV subjects

There were no statistically significant correlations between rCBV values and the inflammation and fibrosis HAI score in HCV infected patients.

### Comparison of rCBV values between HIV-1 and HCV patients and control group (CG) using the Bonferroni correction (significant p<0.0055)

According to the Bonferroni correction HCV subjects showed a significant decrease of rCBV values in left temporoparietal and left frontal cortices compared to CG. This group revealed a significant increase of rCBV value in both basal ganglia regions compared to CG, as well as to HIV-1 naive, HIV-1cART treated and HIV-1/HCV naive patients (table 3).

We also found a significantly decreased rCBV value in HIV-1/HCV naive patients in left temporoparietal cortex.

There were no statistically significant changes between the studied groups in rCBV values in other analyzed regions according to the Bonferroni correction.

## Discussion

In our study we assessed early cerebral perfusion alterations in HIV-1 naive, HIV-1cART treated, HIV-1/HCV and HCV-positive naive patients, who presented a normal appearing brain on plain MR. We found significantly lower rCBV values (p<0.05) in several cortical regions in all HIV-1-positive and HCV-positive patients compared to controls, while there were no significant perfusion alterations in the white matter areas. The HIV-1-positive cART treated patients presented with more pronounced perfusion changes compared to non-treated HIV-1-positive subjects, as the cART treated patients revealed hypoperfusion in more cortical regions. Moreover, HCV-infected patients seemed to reveal the most significant cerebral perfusion changes, as they presented with the significant alterations even with the Bonferroni correction (p<0.0055). Hyperperfusion in basal ganglia in HCV patients is an interesting finding, which we try to explain.

The studied groups were neurologically asymptomatic. There were no significant differences between HIV-1 and HCV-infected patients and the control subjects in executive functions measured with all the WCST scales. We only found that HCV patients performed the d2 attention task significantly slower than healthy controls and had lower values of speed-accuracy variable. Diminished visual concentration endurance and intact executive function suggest that HCV infection is related to specific cognitive disturbances. These disturbances result from changes in brain functioning of a rather focal than generalized nature.

We found the decreased rCBV value within the frontal and temporoparietal cortex bilaterally, as well as in the PCG region in HIV-1-positive and HCV-positive patients. This hypoperfusion of several cortical areas may confirm CNS injury due to HIV-1 as well as HCV infection.

There have been only a few studies which have provided evidence of early cerebral perfusion alterations in neurologically asymptomatic patients with HIV infection [16]–[18] as well as in a macaque model of neuro-AIDS using PWI [23]. Moreover, these studies assessed the perfusion changes in single brain regions, especially in basal ganglia (BG) or in cortical areas. In contrast, we evaluated rCBV values in 9 brain regions including posterior cingulate gyrus (PCG), bilaterally temporoparietal and frontal cortices, parietal white matter areas as well as both BG. Wenserski et al investigated 10 HIV-1-positive patients with normal motor function, and assessed the mean rCBF (regional cerebral blood flow) only in BG and showed no significant perfusion changes compared to the control group [16]. These findings are partially consistent with our results, as we did not observe significant perfusion alterations within the BG regions in HIV-1-positive asymptomatic patients.

On the other hand, Ances et al examined the impact of HIV on rCBF within BG and visual cortex in 33 HIV-1-positive neurologically unimpaired subjects and reported significantly reduced rCBF within BG and visual cortex for both acute/early as well as chronic HIV-infected patients [17]. They concluded that perfusion disorders which occur soon after seroconversion may reflect neuronal or vascular injury of the brain among HIV-infected individuals before they even demonstrate neuropsychological impairment [17].

Our results showing decreased rCBV values in the temporoparietal and frontal cortical regions as well as in PCG are in accordance with previous findings from PET and SPECT studies, which also evidenced hypoperfusion within cortical and subcortical areas in HIV-1 positive patients [24], [25], [18]. Recently, Towgood et al published an interesting paper combining evidence from arterial spin labeling (ASL) and PET method in asymptomatic HIV-1-positive patients. They found significant age effects on both ASL and PET with reduced rCBF and regional cerebral metabolic rate of glucose uptake (rCMRglc) in related frontal brain regions, and consistent, although small, reductions in rCBF and rCMRglc in the anterior cingulate cortex (ACC) in HIV-1 infected patients [18].

The mechanisms for cerebral perfusion disorders observed in the course of HIV infection remain unknown. However, it has been hypothesised that one possible mechanism could be the direct effect of HIV on platelet function [17], [26]. HIV-1-positive individuals demonstrate an increase in the number of activated platelets, which may present increased susceptibility to adhere to each other as well as to blood vessel walls. This process of platelets adhesion stimulates serotonin release and vasoconstriction resulting afterwards in perfusion disturbances. The other possible mechanism may occur due to direct interaction between the virus and endothelial cell migration [17]. Within the brain HIV propagates an increase in cytokine and chemokine release, which could cause deleterious effects to neurons and subsequently may result in perfusion alterations observed in HIV-infected subjects [17]. Another factor contributing to the disturbances in the cerebral microcirculation may be an increased atherosclerosis documented in HIV-1-positive individuals [17], [27].

Furthermore, the studies mentioned above did not evaluate the differences in perfusion alterations between the non-treated and treated HIV-1-positive subjects.

It should be stressed that in our study the HIV-1-positive cART treated patients exhibited more pronounced perfusion changes (decreased rCBV values in 5 brain regions) compared to non-treated subjects who revealed hypoperfusion only in 3 brain areas. We suggest that this could result from prior brain impairment (advanced HIV-1 infection and high viral load) before treatment. Secondly, these changes may be caused by possible neurotoxicity of antiretroviral drugs. The increased frequency of dyslipidemia and glucoregulatory disorders in HIV-positive treated patients has been reported, as well as increased rates of metabolic disorders leading to atherosclerotic disease and associated vascular pathology or increased β-amyloid deposition in the brain [1], [3], [4]. These findings of significantly decreased rCBV values especially in treated subjects are consistent with our previous report showing the most pronounced metabolic disorders in HIV-1-positive cART treated patients [1].

In contrast, as already published in our pilot study, we observed increased rCBV values in the BG region in HCV-infected patients [14]. We hypothesize that the hyperperfusion of BG may be a sign of inflammation in the early stage of HCV brain injury in patients with mild liver disease. This hypothesis corresponds with the data from the literature. Several reports have demonstrated the occurrence of hyperperfusion in HSV encephalitis in PET studies, as well as in other viral CNS infections in SPECT examinations [28]–[31]. Focal hyperperfusion as visualized by SPECT or PET appears to be an indicator of active inflammation of brain tissue [28], [29].

Moreover, it has been reported that the first stage of HIV-1-associated Minor Motor Disorders is characterized by hypermetabolism within the basal ganglia. This hypermetabolism, as assessed with PET, is correlated with hyperperfusion of the BG region in PWI [16], [32].

We would like to point out that HCV-infected patients, although remaining neurologically asymptomatic, were the only subgroup which revealed some subtle differences when performing the d2 attention task compared to controls. This fact may also suggest the brain injury reflected by an inflammation process within BG due to HCV infection similarly to the first stage of HIV-1-associated Minor Motor Disorders [16].

On the other hand, it should be also considered that hyperperfusion in the BG region may indicate the local compensatory increase of perfusion due to hypoperfusion in several cortex areas.

To make the statistical analysis more powerful, we added correction for multiple comparisons. According to the Bonferroni correction there were no statistically significant changes between HIV-1 naive and HIV-1cART treated patients and the control group in rCBV values. However, it should be stressed that we still found significant changes (p<0.0055) in PWI measurements in HCV-infected subjects in the left temporoparietal cortex, the left frontal cortex and in both basal ganglia regions as well as in HIV-1/HCV patients in the left temporoparietal cortex. Our results indicate that HCV-positive and HIV-1/HCV patients with only mild liver disease demonstrate the most pronounced changes in cerebral microcirculation compared to controls as well as to HIV-1 naive and HIV-1cART treated patients. To the best of our knowledge such findings have never been reported before.

Our results of significant cerebral perfusion disturbances in HCV-positive subjects coincide with the current knowledge concerning HCV-related CNS disorders. The spectrum of CNS complications encountered in HCV-positive patients encompasses not only cerebrovascular events but also autoimmune syndromes [12], [13]. The cerebrovascular disorders, which have been documented in HCV-infected individuals included occlusive vasculopathy and vasculitis. Isolated CNS vasculitis has been confirmed by angiographic evidence of multiple focal narrowing of cerebral vessels [13], [33]. Recently, it has been reported that HCV infection could be an independent risk factor for increased carotid wall thickness and plaque formation, which contribute to significant cerebrovascular mortality in HCV-positive subjects [33]. However, the exact mechanism responsible for the CNS vasculitis process related to HCV infection remains poorly understood. It has been hypothesised that recurrent precipitation of cryoglobulins with complement fixation and direct complement pathway activation by HCV itself, might be involved in ischemic and inflammatory tissue damage [12], [13]. On the other hand, the vasculitic process in cryoglobulin-negative HCV individuals, as in our study, may be probably secondary not only to the direct complement activation but also to an interaction between the virus present within the CNS and the host immune system [12].

Additionally, we investigated the correlations between rCBV values and the immunologic data (CD4a and Cd4n T cell count) in HIV-1-positive patients as well as the inflammation and fibrosis HAI score in HCV infected patients. However, we did not find any statistically significant correlations. In the other studies, mentioned above, concerning perfusion changes in asymptomatic HIV-1-positive individuals, the authors did not analyse the possible associations between PWI measurements and the immunologic data. Chang et al reported that rCBF abnormalities correlated significantly with clinical disease severity as measured by CD4 count, plasma viral load, Karnofsky score, and HIV dementia scale, but they evaluated patients with HIV-cognitive motor complex, who presented with the mean AIDS dementia complex (ADC) stage ranging from 0.5 to 2 (moderate dementia) [34]. In contrast, we enrolled in our study only HIV-1-positive asymptomatic patients with ADC stage 0.

Furthermore, except for our previous pilot study concerning early cerebral changes in HCV-positive patients [14], there were no other papers reporting perfusion cerebral alterations in that group of subjects and no other possible correlations between PWI measurements and the inflammation and fibrosis HAI score in HCV infected patients have been published in the literature.

## Conclusion

Perfusion-weighted MR examination is capable of depicting cerebral perfusion alterations in neurologically asymptomatic HIV-1-positive patients in the early stage of disease before progressing to HIV-associated neurocognitive disorders (HAND) as well as in HCV-positive patients with only mild liver disease. The HIV-1-positive cART treated patients presented with more pronounced perfusion changes compared to non-treated HIV-1-positive subjects. Moreover, HCV-infected patients seemed to reveal the most significant cerebral perfusion changes. Hyperperfusion in basal ganglia may be an indicator of brain inflammation in HCV patients.

In our opinion rCBV measurement could be a noninvasive neuroimaging biomarker for assessing early cerebral microcirculation impairment in HIV-1 as well as in HCV infections.
